# *Leptospira* Seroprevalence in Bardigiano Horses in Northern Italy

**DOI:** 10.3390/ani10010023

**Published:** 2019-12-20

**Authors:** Elena Vera, Simone Taddei, Sandro Cavirani, Jennifer Schiavi, Mario Angelone, Clotilde S. Cabassi, Emiliana Schiano, Fausto Quintavalla

**Affiliations:** Department of Veterinary Science, University of Parma, via del Taglio 10, 43126 Parma, Italy; elenavera.dvm@gmail.com (E.V.); sandro.cavirani@unipr.it (S.C.); jennifer.schiavi93@gmail.com (J.S.); marioangelonevet@libero.it (M.A.); clotildesilvia.cabassi@unipr.it (C.S.C.); emiliana.schiano@unipr.it (E.S.); fausto.quintavalla@unipr.it (F.Q.)

**Keywords:** Bardigiano, horse, leptospirosis, risk factors, seroprevalence

## Abstract

**Simple Summary:**

Leptospirosis is one of the most widespread zoonoses worldwide and is considered a re-emerging disease. In horses, leptospiral infection frequently does not result in a systemic disease and it is commonly believed that horses play a minor role in spreading the disease, compared to other livestock and wild animals. However, horses can become carriers and it has been suggested that the horse is the maintenance host for serovar Bratislava. Epidemiological data regarding leptospirosis in horses in Europe are lacking and further studies are required. The aim of this study was to evaluate *Leptospira* seroprevalence in Bardigiano horses living in the Province of Parma, Northern Italy, and to identify risk factors associated with seropositivity. A high seroprevalence against *Leptospira* spp. among Bardigiano horses and a high number of infected farms were found. Unexpectedly, seroprevalence was considerably higher compared to similar studies carried out in Italy. The location of the farm and the type of housing did not affect seroprevalence, but rodent control might reduce the risk of exposure for Bardigiano horses. Horses living in the considered area have high-risk exposure to different serovars of pathogenic leptospires and could contribute to the maintenance of the bacterium in the environment.

**Abstract:**

A cross-sectional study was carried out in Bardigiano horses in the Province of Parma, Northern Italy, to assess the seroprevalence of *Leptospira* spp. and to investigate risk factors associated with the infection. A representative sample of 134 horses from 43 farms was selected by stratified systematic randomization. Blood sera were examined by MAT for the presence of antibodies against seven *Leptospira* serovars. Ninety animals (67.2%; 95% Confidence Interval 63.2–71.1) and 41 farms (95.3%; 95% CI 92.2–98.5%) were found positive to at least one of the serovars. The most frequently detected reactions were against serovar Bratislava (41.8%), followed by Canicola (36.6%), Tarassovi (28.4%), Copenhageni (17.9%), Pomona (10.4%) and Hardjo (2.2%). None of the sera reacted against serovar Grippothyphosa. Forty-eight horses (53.3% of the seropositives) were positive for more than one serovar and 21 (15.7% of the seropositives) had serum titres ≥ 1000. Bratislava was the serovar providing the highest antibody titres. Prevalence was significantly higher between adult horses and in farms lacking rodent control (*p* = 0.006 and *p* = 0.025, respectively). No significant gender or housing-related difference in seroprevalence was found. The anamnestic data suggest that the infection in Bardigiano horses is subclinical in most of the cases. The high seroprevalence indicates that Bardigiano horses living in the investigated area are at high risk of exposure and infection by *Leptospira* spp.

## 1. Introduction

Leptospirosis is caused by spirochetes belonging to the genus *Leptospira* (family Leptospiraceae, order Spirochaetales) and is one of the most widespread zoonoses worldwide [1,2]. Wild animals, livestock and pets can act as reservoirs of the bacterium. In Italy, reporting of cases of this disease is mandatory with this zoonoses being monitored according to the country’s epidemiological situation (Directive 2003/99/EC). However, due to a non-specific clinical picture, leptospirosis is often not recognized and widely underestimated in both humans and animals. Therefore, laboratory tests are paramount for diagnosis [3].

As recently suggested [4], epidemiological data regarding leptospirosis in horses in Europe are lacking. In horses, leptospirosis does not commonly cause systemic disease [5]. Infection during pregnancy can result in placentitis, abortion, stillbirths or neonatal mortality, birth of weak foals [6]. Renal localization of serovar Pomona occasionally causes fever and acute renal failure, especially in foals [5]. The classic icteric form of leptospirosis could be observed in young animals, whereas it is not commonly reported in adult horses. Moreover, *Leptospira* spp. is considered as the most common infectious cause of equine recurrent uveitis (ERU) [5]. Respiratory disorders may also occur [7,8].

The Bardigiano is an ancient horse breed from the Northern Apennine region of Italy. The first information on the Bardigiano horse dates back to 1864 and the origin of the breed can be traced to the horse of Belgian Gaul [9]. The breed takes its name from the medieval village of Bardi, located in the Province of Parma, and consists of a homogeneous population of horses with typical and distinct traits. The Bardigiano horse falls into the pony category. The breed is meso-brachymorphic type and the coat color is bay, with dark bay being the most prevalent. The traditional use of Bardigiano horse, long since appreciated for its rusticity and docility, was that of agricultural work in mountain areas, besides meat production. However, in recent decades, the Bardigiano horse was also confirmed to be very suitable for use as a saddle horse, especially for tourism purposes, and for pet therapy. Bardigiano horse breeding is widespread in the Province of Parma and in hilly and mountainous areas of the Regions Emilia-Romagna, Liguria and Tuscany. However, this breed is spreading to other Italian regions, as well as to other European nations, such as Germany and Hungary. In Italy, the current population is estimated at 3500 Bardigiano horses (data from the Ministry of agricultural food, forestry and tourism policies. Available online: https://www.politicheagricole.it/flex/cm/pages/ServeBLOB.php/L/IT/IDPagina/6179 (accessed on 17 September 2019)), with the Province of Parma accounting for about 700 animals. Most of them are kept out on pasture, being stabled during the winter only.

The aim of this study was to evaluate the seroprevalence of *Leptospira* in Bardigiano horses living in the Province of Parma and to identify risk factors associated with seropositivity.

## 2. Materials and Methods

### 2.1. Horses

**Figure 1:**
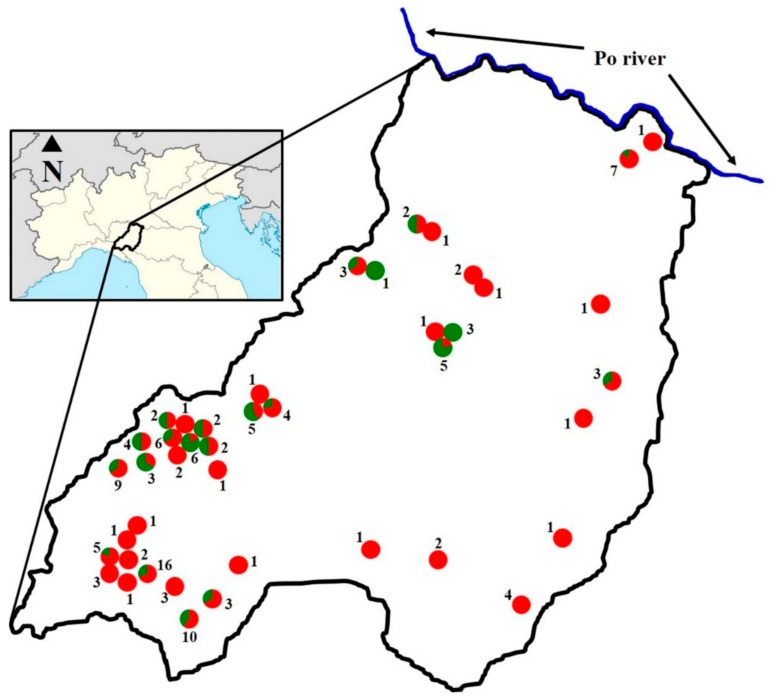
Location of farms. The province of Parma is highlighted by the black outline. Each farm is indicated by a circle. The number of tested animals in each farm is indicated by Arabic numerals. The proportion of positive and negative animals among the horses tested in each farm is shown in red and green, respectively.

The study was carried out in 2016–2017 on 134 Bardigiano horses living in 43 different farms of the Province of Parma (Figure 1). 

The farms were assigned to two different groups. The ‘mountain’ group included the farms located in the municipalities of Albareto, Bardi, Bedonia, Berceto, Borgo Val di Taro, Compiano, Corniglio, Monchio delle Corti, Palanzano, Tornolo and Varsi. The ‘valley’ group included the farms located in the municipalities of Colorno, Fidenza, Lesignano de’ Bagni, Medesano, Montechiarugolo, Noceto, Parma, Salsomaggiore Terme and Salsominore. The altitude of the farms in the valley area ranged from 25 to 290 m above sea level (mean ± standard deviation: 128 ± 75) and that of the farms in the mountain area ranged from 440 to 1050 m above sea level (666 ± 154).

None of the animals were vaccinated against leptospirosis. The sample size required to estimate the prevalence of *Leptospira* in horses was determined to be at least 132 horses (population size 700, data provided by the breeders’ association of the Province of Parma). The expected prevalence was 12%. The value of the expected prevalence was established on the basis of the higher leptospirosis seroprevalence value reported in surveys carried out on horses in Italy and available at the time of study planning [10,11]. The accuracy and confidence level were 5% and 95%, respectively. Animals were selected by stratified systematic randomization. Stratification was based on the type of housing.

### 2.2. Sampling

Blood samples were collected by venipuncture of the jugular vein into 10 mL tubes (Vacutainer, Becton Dickinson) without anticoagulant, kept at refrigeration temperature and delivered to the laboratory within the same day. Each serum was immediately separated by centrifugation at 1000× *g* and stored at −20 °C until it was analyzed. Blood sampling was conducted in compliance with national (Decreto Legislativo n. 26/2014, art. 2) and European (Directive 2010/63/EU) laws and policies regarding the protection of animals used for experimental and other scientific purposes. Moreover, the present project was approved by the Ethical Committee of the University of Parma (Organismo Preposto al Benessere degli Animali—prot. n. 04/CE/2019). For each animal, an anamnestic form was completed at the time of sampling to obtain information regarding horse’s sex, age, medical history and health status (with particular reference to the clinical signs attributable to leptospirosis, such as abortion and still births, uveitis, fever, kidney disease and jaundice), in addition to farming conditions. The form also contained questions concerning the presence of other domestic or wild animals that could come in contact with the horses, either directly or through the sharing of grazing pastures. Finally, the presence of integrated pest control management was investigated.

### 2.3. Serology

A microscopic agglutination test (MAT) was performed using a panel of seven *Leptospira* serovars (serogroup Australis, serovar Bratislava; serogroup Canicola, serovar Canicola; serogroup Icterohaemorrhagiae, serovar Copenhageni; serogroup Grippotyphosa, serovar Grippotyphosa; serogroup Sejroe, serovar Hardjo; serogroup Pomona, serovar Pomona; serogroup Tarassovi, serovar Tarassovi). *Leptospira* strains were cultured in liquid EMJH medium (Becton Dickinson) for three–four days at 30 °C (to a density of approximately 2–4 × 10^8^ leptospires per mL) and diluted 1:2 in sterile saline. All sera were first screened at 1:100 dilution by adding 150 μL of the diluted *Leptospira* suspensions to 25 μL of each serum previously diluted 1:14 in sterile saline. After four hours incubation at 37 °C [12], 8–10 μL of each suspension was transferred on a slide and examined under a dark field microscope (Eclipse 50i, Nikon) at 100× magnification. Sera that gave a positive reaction were further titrated in serial two-fold dilutions, starting from 1:125 to titre end-point. Antibody titres were expressed as the reciprocal of the highest dilution of serum that gave 50% or more of reduction of free leptospires in the suspension, compared to a negative control obtained by using sterile saline. A positive reaction of a serum against all serovars would be considered non-specific. A titre ≥ 100 was deemed positive, i.e., indicating *Leptospira* exposure or infection.

### 2.4. Statistical Analysis

Statistical analysis was performed using the chi-squared test. *p* values lower than 0.05 were regarded as statistically significant.

## 3. Results

Ninety horses from 41 different farms exhibited positive MAT titres to one or more serovars of *Leptospira* at a serum dilution of 1:100. This means that 67.2% (95% Confidence Interval 63.2–71.1) of the animals and 95.3% (95% CI 92.2–98.5%) of the farms were positive. Clinical signs compatible with leptospirosis were recorded in two horses. One presenting uveitis and the other one having had an abortion. The horse with uveitis was seronegative, while the animal who had an abortion was positive to serovars Bratislava and Canicola. The highest number of positive animals was found for the Bratislava serovar, followed by Canicola, Tarassovi, Copenhageni, Pomona and Hardjo (Table 1). No animals were found positive for the serovar Grippotyphosa.

Forty-six point seven percent (42/90) of the seropositive horses reacted against a single serovar and 53.3% (48/90) was found to be positive to multiple serovars (Table 2). 

**Figure 2:**
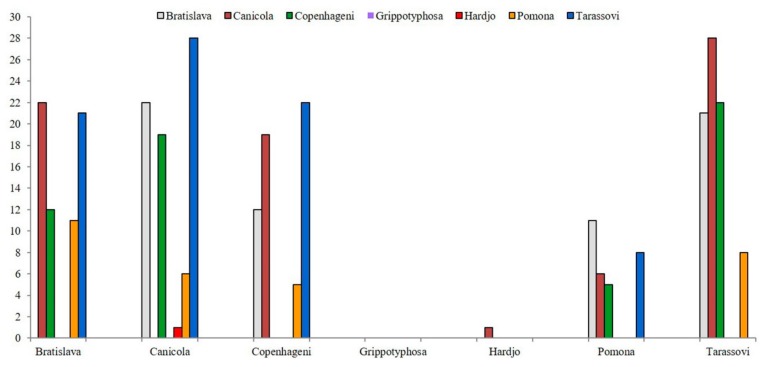
Multiple seropositivity. The number of positive animals for each pair of serovars is reported. Serovar pairs are indicated by the name on the abscissa and the color of the bar.

The highest frequency of multiple positivity was detected against the pair of serovars Canicola and Tarassovi, followed by Bratislava–Canicola and Copenhageni–Tarassovi pairs, which showed the same value (Figure 2).

**Figure 3:**
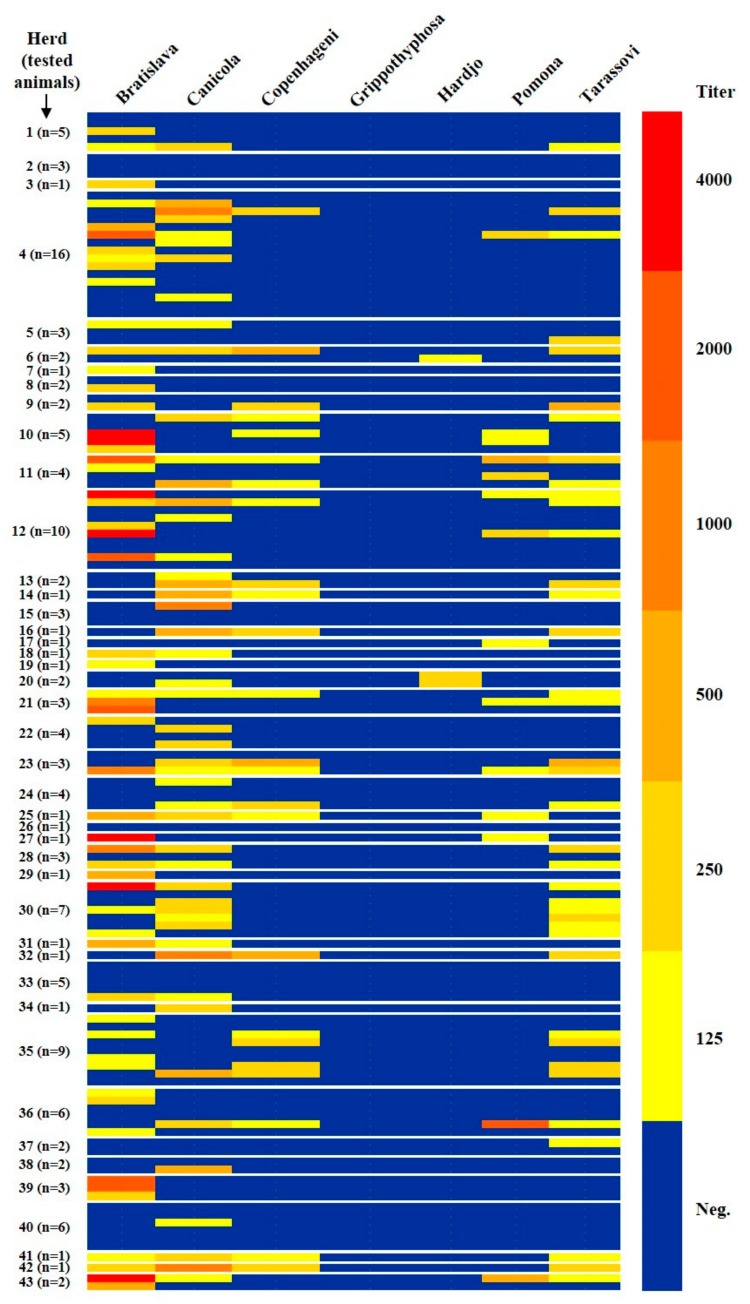
Heat map of seropositivity to the different serovars.

Most of the MAT titres were relatively low. The highest titer was 4000 and the modal titer was 125 (Table 1 and Figure 3). Among the seropositives, 21/134 (15.7%) showed antibody titres greater than or equal to 1000. All the seropositives with antibody titres greater than or equal to 1000 were positive to a single serovar. In particular, 16 towards Bratislava, four towards Canicola and one towards Pomona. The Bratislava serovar was also the one that gave the highest antibody titres: out of 16 Bratislava positive sera with values ≥1000, seven reacted with antibody titer equal to 4000 (Table 1 and Figure 3).

The results for potential risk factors are shown in Table 3.

A statistically significant higher seroprevalence was detected with increasing age (*p* = 0.011). No significant difference of seroprevalence was detected between 6–15 and 16–30 year-old animals (*p* = 0.205). Statistical significance further increases (*p* = 0.006) by comparing the group of all adult animals (6–30 years old) with the group of young animals (1–5 years old). Similarly, a significantly higher seroprevalence (*p* = 0.025) was found for horses belonging to farms without rodent control programs compared to those belonging to farms in which preventive measures against rodents were taken. Comparing free ranging horses with those kept in a box or paddock, no statistically significant difference was observed (*p* = 0.724). The different seroprevalence between horses within the ‘mountain’ group and ‘valley’ group was not statistically significant (*p* = 0.520). The presence of domestic or wild animals did not significantly affected seroprevalence (*p* = 0.937 and *p* = 0.425, respectively). Regarding sex, no significant difference between males and females was found, counting stallions and geldings separately (*p* = 0.542) or grouping them together (*p* = 0.726).

## 4. Discussion

The prevalence and impact of leptospirosis in horses remain unclear, especially in European countries [4]. The incidence of the disease and the serovars involved vary according to the geographical area [5,13]. Horses are not commonly considered a possible source of leptospirosis diffusion compared to other livestock and wild animals. However, horses may harbor leptospires in the kidney, becoming carriers and causing the spread of the bacterium in the environment [6]. The present study assessed, using the MAT, the prevalence of antibodies against seven serovar of *Leptospira* in Bardigiano horses living in the Province of Parma. We found that 67.2% of the animals exhibited positive MAT titres to at least one serovar and 53.3% of positive horses had antibodies against more serovars. Seropositivities to more serovars may be due to multiple infections or to cross-reactivities. Since all the horses involved were not vaccinated against leptospirosis, we concluded that the detection of antibodies was indicative of *Leptospira* exposure or infection. The high seroprevalence of *Leptospira* spp. found in Bardigiano horses is in agreement with other Europeans reports. Seroprevalences of 58.5% and 79% were found in Switzerland and in the Netherlands, respectively [14,15]. In a Brazilian study, the percentage of seropositivity was 71.4% [16] and a recent study carried out in some States of the American Midwest reported a 77% seroprevalence in healthy horses [17]. Surprisingly, a group of Italian researchers [10,11] reported a seroprevalence of 11.4% and 1.5%, respectively. Ebani et al. (2012) ascribed the results of their study to the type of the environment. The horses included in their study lived in areas with a low presence of stagnant water and under good management conditions. The hydrologic density is recognised to be a risk factor [18]. The territory of the Province of Parma belongs to a geographical area different from the one considered by Ebani et al. (2012). In fact, it is included in the Padanian hydrographic district, which is characterized by rainfall and water runoff values higher than all other Italian hydrographic districts (report of the National Statistical Institute—Giornata mondiale dell’acqua, 2015. Available online: https://www.istat.it/it/files//2015/03/Statistiche-sullacqua.pdf).

The considered territory is crossed by several rivers and numerous channels for water flow regulation. Moreover, the climate of the Po Valley is characterized by high humidity compared to other part of Italy. Furthermore, in the study of Cerri et al. (2003), a cut-off of 400 was used to consider a serum positive and this reduces the prevalence value compared to a cut-off of 100. A 400 cut-off is useful to reduce possible cross-reactions and vaccinal antibodies interference [19]. However, in this study, antibody interference by vaccination could be excluded as none of the horses were vaccinated against leptospirosis. Several years later, another Italian study reported a seroprevalence of 29.7% with a 100 cut-off value [19]. This last study was a nationwide serological survey involving 747 horse sera collected by all 10 Italian Istituti Zooprofilattici Sperimentali (IIZZSS) between 2010 and 2011. Another very recent study reported a seroprevalence of 2.89% with a 100 cut-off value [20]. In this case, only 74 horse sera collected in North-Central Italy in the period 2002–2016 were tested. In accordance with literature data [10,14,19,21,22], in this study, Bratislava was the serovar showing the highest prevalence of MAT positive reactions. Serovar Bratislava is considered by most researchers to be the host-adapted serovar in the horse and horses may also act as maintenance hosts [21,22,23,24]. Twenty-one horses exhibited antibody titres ≥1000 to serovars Bratislava, Canicola and Pomona. Bratislava was the serovar with the highest MAT titres. It might be unusual for an host-adapted serovar, but not completely unexpected [22]. In endemic areas, titres ≥ 800–1600 in the presence of compatible symptoms may be considered indicative of leptospirosis [25]. All horses involved in this study did not show clinical signs attributable to leptospirosis, except for two subjects in which uveitis and abortion were reported. In both cases, the origin of the disorder could not be established. The horse with uveitis was seronegative to all serovars tested. However, the involvement of *Leptospira* cannot be excluded since it has been proved that serology alone may not be able to diagnose *Leptospira*-associated uveitis [4,26,27,28]. Interestingly, there were no seropositive animals for serovar Grippotyphosa. While this serovar occurs sporadically in horses, it is considered to be the most common serovar associated with ERU in Europe [29]. The horse that aborted was seropositive and antibody titres were 500 against Bratislava and 125 against Canicola. Therefore, the involvement of one of these serovars in that abortion cannot be excluded. The results are thus in agreement with those reported by different authors: seropositive or infected horses are, in most cases, asymptomatic [5,22].

Our data show a significatively higher seropositivity to *Leptospira* spp. in adult horses compared to young horses. This result is in agreement with data reported by other authors [15,21,24] and could be explained considering that the probability of coming into contact with *Leptospira* increases with increasing age and that the seropositivity can persist for a long time. However, other authors have found no significant association between seropositivity and age [30]. Contrarily to what was reported by other studies [15,21,30,31], in this survey, sex differences were not significantly associated with seropositivity. The Province of Parma is characterized by mountains to the south-west and plains to the north-east. Free-ranging horses cannot pass from one area to the other, due to the anthropization of the territory which limits the movement of these animals. Therefore, the location of the farms was also evaluated as a potential risk factor. The location of farms in the valley or in the mountain areas did not significantly affect seroprevalence values. This result could be partly explained by the fact that, despite the orographic differences, all the territory of the Province of Parma is in the same hydrographic area. All the rivers that cross the territory come from the Apennine range and flow in a north-east direction up to the Po river. It is interesting to note that only two of the 41 farms included in the study were completely negative, suggesting a wide dissemination of pathogenic leptospires in the considered area. Blatti et al. (2011) have hypothesized that the higher seroprevalence detected in ponies compared to horses could be related to the longer time spent grazing by ponies. A similar hypothesis was made by other authors to explain the higher prevalence of leptospiral antibody rate in donkeys compared to horses [32]. However, this study shows that in Bardigiano horses, the risk of infection is the same regardless of the time the animal spent grazing, turned out or housed in the stable during the year. The role of the type of housing in *Leptospira* spp. transmission could be hidden by the presence or absence of rodents, which represent a major risk factor for *Leptospira* prevalence [6,25]. Rodents are probably the predominant type of wildlife in the horses’ indoor environment and the density of the rat population was positively associated with the prevalence of *Leptospira interrogans* [33]. The presence of pest control measures significantly reduced the chance for Bardigiano horses getting the infection. Although the role of rodents in transmission of leptospiral serovars can sometimes be difficult to evaluate [31], rodent control is considered an important factor for prevention [6,34] and our data confirm this notion. In this study, the presence of other domestic animals did not significantly affect the seroprevalence. Domestic animals were mostly dogs, cats and poultry. Pigs act as maintenance hosts of serovar Pomona [35]. Their presence was reported in only one farm with the horse living there being negative for all tested serovars. On the other hand, all Bardigiano horses positive to serovar Hardjo, whose maintenance host is cattle [36], were nearby or in a cattle farm.

## 5. Conclusions

This study revealed a high seroprevalence against *Leptospira* spp. among Bardigiano horses and a high number of infected farms. Seroprevalence was considerably higher compared to similar studies carried out in Italy [10,11,19]. Horses living in the considered area, therefore, have high risk exposure to pathogenic leptospires. However, anamnestic data suggested that in Bardigiano horses, the infection is mostly subclinical. In agreement with other authors [33], this study showed how improving some management practices, especially rodent control, might reduce the risk of exposure for horses and hopefully, for humans.

## Figures and Tables

**Table 1:** Distribution of MAT antibody titres for each *Leptospira* serovar.

Serovar	Number of Positive Animals for Each Antibody Titre						Number of Positive Animals/Overall (%)
	125	250	500	1000	2000	4000	
Bratislava	18	17	5	3	6	7	56/134 (41.8)
Canicola	20	17	8	4	0	0	49/134 (36.6)
Copenhageni	12	9	3	0	0	0	24/134 (17.9)
Grippotyphosa	0	0	0	0	0	0	0/134 (0)
Hardjo	1	2	0	0	0	0	3/134 (2.2)
Pomona	8	3	2	0	1	0	14/134 (10.4)
Tarassovi	22	14	2	0	0	0	38/134 (28.4)
Total	81	62	20	7	7	7	

**Table 2:** Number of animals with single or multiple seropositivity.

Number of Serovars to Which the Animal was Positive	Animals/Overall Number of Positives (%)
1	42/90 (46.7)
2	15/90 (16.7)
3	22/90 (24.4)
4	9/90 (0.1)
5	2/90 (2.2)
6	0/90 (0)
7	0/90 (0)

**Table 3:** Distribution of seroprevalence for *Leptospira* spp. by demographic, geographic and management factors.

Potential Risk Factor	Tested Animals	Positive Animals	Seroprevalence (%)	95% CI ^1^
Age				
1–5 years	18	7	38.9	27.6–50.1
6–15 years	93	69	74.2	69.7–78.6
16–30 years	23	14	60.9	50.9–70.8
Sex				
Female	97	66	68.0	63.4–72.7
Male	24	17	70.8	61.7–79.9
Gelding	13	7	53.8	40.3–67.4
Location of the farm				
Mountain	102	70	68.6	64.1–73.1
Valley	32	20	62.5	54.1–70.9
Rodent control				
Yes	52	29	55.8	49.0–62.5
No	82	61	74.4	69.7–79.1
Housing				
Box or paddock	34	22	64.7	56.7–72.7
Free ranging	100	68	68.0	63.4–72.6
Presence of other domestic animals ^2^				
Yes	95	64	67.4	62.7–72.1
No	39	26	66.7	59.3–74.1
Presence of wild animals ^3^				
Yes	85	55	64.7	59.6–69.8
No	49	35	71.4	65.1–77.8

^1^ Confidence interval. ^2^ Reported domestic animals: cats, dogs, goats, poultry and pigs. ^3^ Reported wild animals: deer, foxes, roes, wolves, wild boars.
